# Clinical Characteristics and Treatment Outcomes of Myeloid Sarcoma in Children: The Experience of the Polish Pediatric Leukemia and Lymphoma Study Group

**DOI:** 10.3389/fonc.2022.935373

**Published:** 2022-07-07

**Authors:** Magdalena Samborska, Małgorzata Barańska, Jacek Wachowiak, Jolanta Skalska-Sadowska, Sheanda Thambyrajah, Małgorzata Czogała, Walentyna Balwierz, Sylwia Kołtan, Katarzyna Peszyńska-Żelazny, Mariusz Wysocki, Tomasz Ociepa, Tomasz Urasiński, Grażyna Wróbel, Jadwiga Węcławek-Tompol, Bogna Ukielska, Alicja Chybicka, Anna Kitszel, Maryna Krawczuk-Rybak, Anna Szmydki-Baran, Iwona Malinowska, Michał Matysiak, Agnieszka Mizia-Malarz, Renata Tomaszewska, Tomasz Szczepański, Agnieszka Chodała-Grzywacz, Grażyna Karolczyk, Lucyna Maciejka-Kembłowska, Ninela Irga-Jaworska, Wanda Badowska, Michał Dopierała, Paweł Kurzawa, Katarzyna Derwich

**Affiliations:** ^1^ Department of Pediatric Oncology, Hematology and Transplantology, Poznań University of Medical Sciences, Poznań, Poland; ^2^ Department of Pediatric Oncology and Hematology, University Children’s Hospital, Kraków, Poland; ^3^ Department of Paediatrics, Hematology and Oncology, Collegium Medicum in Bydgoszcz, Nicolaus Copernicus University in Toruń, Toruń, Poland; ^4^ Department of Pediatrics, Hemato-oncology and Pediatric Gastroenterology, Independent Public Clinical Hospital No. 1, Pomeranian Medical University, Szczecin, Poland; ^5^ Department of Bone Marrow Transplantation, Pediatric Oncology and Hematology, Supraregional Center of Pediatric Oncology “Cape of Hope”, Wrocław, Poland; ^6^ Department of Pediatrics, Oncology and Hematology, L. Children’s Clinical Hospital, Białystok, Poland; ^7^ Department of Pediatric Oncology and Hematology, Independent Public Children’s Teaching Hospital, Warsaw, Poland; ^8^ Department of Oncology, Hematology and Chemotherapy, John Paul II Upper Silesian Child Health Centre, Katowice, Poland; ^9^ Department of Pediatrics, Pediatric Hematology and Oncology in Zabrze, Stanisław Szyszko Independent Public University Hospital No. 1, Medical University of Silesia, Katowice, Poland; ^10^ Department of Pediatric Oncology and Hematology, Provincial Integrated Hospital, Kielce, Poland; ^11^ Department of Pediatrics, Hematology and Oncology, University Clinical Centre, Gdańsk, Poland; ^12^ Department of Pediatric Oncology and Hematology, Provincial Specialist Children’s Hospital, Olsztyn, Poland; ^13^ Department of Clinical Pathology, Poznań University of Medical Sciences, Poznań, Poland

**Keywords:** children, myeloid sarcoma, acute myeloid leukemia, prognosis, treatment

## Abstract

**Introduction:**

Myeloid sarcoma (MS) is an extramedullary malignant tumor composed of immature myeloid cells. It occurs in patients with acute myeloid leukemia (AML), myelodysplastic syndrome (MDS), or chronic myeloid leukemia (CML). MS may coincide with disease diagnosis or precede bone marrow involvement by months or even years; it can also represent the extramedullary manifestation of a relapse (1, 2).

**Aim:**

The aim of this study is to describe clinical characteristics of children diagnosed with MS in Poland as well as to analyze diagnostic methods, treatment, and outcomes including overall survival (OS), relapse-free survival (RFS), and event-free survival (EFS). The study also attempted to identify factors determining treatment outcomes.

**Patients:**

The study group comprised 43 patients (F=18, M=25) aged 0-18 years (median age, 10.0 years; mean age, 8.8 years) diagnosed with MS based on tumor biopsy and immunohistochemistry or identification of underlying bone marrow disease and extramedullary tumor according to imaging findings.

**Methods:**

The clinical data and diagnostic and therapeutic methods used in the study group were analyzed. A statistical analysis of the treatment outcomes was conducted with STATISTICA v. 13 (StatSoft, Inc., Tulsa, OK, USA) and analysis of survival curves was conducted with MedCalc 11.5.1 (MedCalc Software, Ostend, Belgium). Statistical significance was considered at p<0.05.

**Results:**

In the study group, MS was most frequently accompanied by AML. The most common site of involvement was skin, followed by orbital region. Skin manifestation of MS was more common in the age group <10 years. The most frequent genetic abnormality was the t(8;21)(q22;q22) translocation. The 5-year OS probability (pOS), 5-year RFS probability (pRFS), and 5-year EFS probability (pEFS) were 0.67 ± 0.08, 0.79 ± 0.07, and 0.65 ± 0.08, respectively. In patients with isolated MS and those with concurrent bone marrow involvement by AML/MDS, pOS values were 0.56 ± 0.12 and 0.84 ± 0.09 (p=0.0251), respectively, and pEFS values were 0.56 ± 0.12 and 0.82 ± 0.08 (p=0.0247), respectively. In patients with and without the t(8;21)(q22;q22) translocation, pEFS values were 0.90 ± 0.09 and 0.51 ± 0.14 (p=0.0490), respectively.

**Conclusions:**

MS is a disease with a highly variable clinical course. Worse treatment outcomes were observed in patients with isolated MS compared to those with concurrent bone marrow involvement by AML/MDS. Patients with the t(8;21)(q22;q22) translocation were found to have significantly higher pEFS. MS location, age group, chemotherapy regimen, surgery, and/or radiotherapy did not have a significant influence on treatment outcomes. Further exploration of prognostic factors in children with MS is indicated.

## Introduction

According to the World Health Organization (WHO) 2008 classification, myeloid sarcoma (MS) is categorized as an acute myeloid leukemia (AML)–related neoplasm. It results from proliferation of cells of one or more myeloid lineages that subsequently form an extramedullary tumor mass disrupting the surrounding tissue architecture. MS was firstly described by Burns in 1811, followed by King in 1853, who used the term chloroma due e to the green appearance of cells under microscopic imaging as a result of the presence of myeloperoxidase (1, 2). Other names used in past literature are granulocytic sarcoma, extramedullary myeloid cell tumor, and myeloblastoma (3, 4).

The etiology of MS remains unclear. One of the factors promoting blast survival in extramedullary tissues is the presence of natural barriers (blood-brain barrier and blood-testis barrier) that impede the penetration of chemotherapeutic agents to the central nervous system (CNS) and gonads (5). A variety of mechanisms are suspected to underlie enhanced blast migration to extramedullary tissues, including the formation of complexes of metalloproteinases and leukocyte integrins (6, 7) as well as interactions between specific chemokines and their receptors (8).

MS most frequently coincides with a diagnosis of AML; it presents concurrently with AML in 2-8% of adult patients and up to 40% of pediatric patients (3, 5). However, statistical data of pediatric patients are limited. An analysis by Kobayashi et al. reported that among 240 patients with AML, 23.3% had extramedullary disease at baseline. The study also included patients with MS with CNS involvement (9). An analysis by Dusenbery et al. showed that extramedullary manifestation at diagnosis was found in 10.9% of 1,832 patients (10). In a study by Johnston et al., MS was diagnosed in 99 of 1,459 patients (6.7%) (11).

Isolated MS without concurrent bone marrow involvement (*de novo* MS) poses certain diagnostic problems. In children, only ambiguous cases of isolated MS have been reported. According to the literature, the incidence of isolated MS is 2/1,000,000 in adults and 0.7/1,000,000 in children. Untreated isolated MS progresses to AML in 90% of cases and mean time from diagnosis to bone marrow involvement is approximately 10.5-11 months (4).

A major issue is an extramedullary relapse of MS experienced by 5-12% of patients after allogeneic hematopoietic stem cell transplantation (alloHSCT) (12). The factors that are likely to increase the risk of such a relapse include the presence of extramedullary locations in the disease course, AML M4 or M5 according to the French–American–British (FAB) classification, advanced disease at transplantation, and high-risk genetic factors such as t(8;21), inv(16) and KMT2A rearrangement (12–15).

### Aim

A summary of the experience of Polish pediatric oncology centers regarding pediatric patients with MS is an important step towards a deeper understanding of this rare disease. The aim of this study is to describe clinical characteristics of children diagnosed with MS in Poland as well as to analyze diagnostic methods, treatment, and outcomes, including overall survival (OS), relapse-free survival (RFS), and event-free survival (EFS). The study also seeks to identify factors influencing treatment outcomes.

## Methods

An Excel database was created to collect data on clinical features and treatment methods and outcomes. An event was defined as a relapse, progression, death, or second cancer. Complete remission (CR), partial remission (PR), and late remission (LR) were assessed according to the patient’s AML therapeutic protocol. Moreover, PR was reported in the case of partial tumor regression, even if CR in the bone marrow was achieved.

Qualitative variables were presented as numbers (N) and percentages (%). For bivariate comparisons, χ^2^ or the Fisher’s exact test was applied. χ^2^ test was also used for multivariate comparisons.

Quantitative variables were expressed as mean ± standard deviation (SD), minimum and maximum values, and median. Normal distribution of the variables was assessed by performing the Shapiro-Wilk test. Since most of the variables did not follow Gaussian distribution, all the analyses were conducted using non-parametric tests. Comparisons between the two groups were performed using the non-parametric Mann–Whitney U test for independent variables.

The Kaplan–Meier method was used to determine pOS, pRFS, and pEFS. The influence of various categories (age group, MS presentation, tumor location, t(8;21)(q22;q22) translocation, treatment regimen, alloHSCT, surgical treatment, and radiotherapy) on survival times (OS, EFS, RFS) was assessed using the logrank test.

A statistical analysis was conducted with STATISTICA v. 13 (StatSoft, Inc, Tulsa, OK, USA) and analysis of survival curves was conducted with MedCalc 11.5.1 (MedCalc Software, Ostend, Belgium). Statistical significance was considered at p<0.05.

### Patients

The study group comprised 43 patients with MS (F=18, M=25) aged 0-18 years (median, 10.0 years; mean, 8.8 years) who were hospitalized in 12 pediatric oncology and hematology centers in Poland between 1998 and 2019. Inclusion criteria were as follows: age from 0 to 19 years and diagnosis of MS based on histopathology and immunohistochemistry or clinical presentation in those patients with an extramedullary tumor and concurrent proliferative disease in the bone marrow such as AML, myelodysplastic syndrome (MDS), or chronic myeloid leukemia (CML).

For research purposes, patients were divided into two age groups: <10 years (n=21) and ≥10 years (n=22).

## Results

### 1. Study Group Characteristics

#### Diagnosis of Underlying Disease and Clinical Presentation of MS

In the study group, patients with AML predominated (29), twelve patients had isolated MS without bone marrow involvement, one patient was diagnosed with CML, and one patient was diagnosed with MDS.

**Figure 1:**
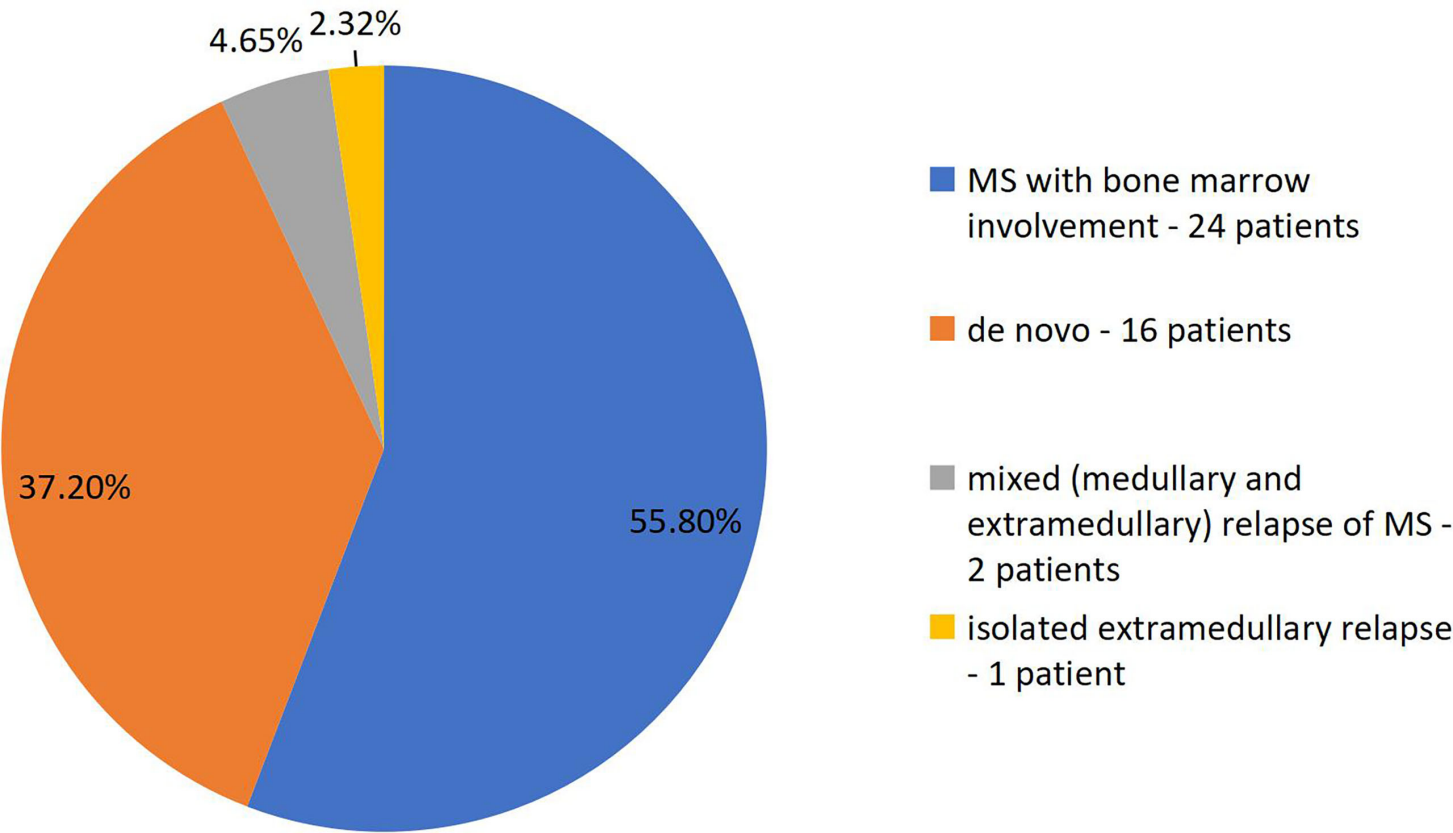
Clinical presentations of myeloid sarcoma depending on the time relation to bone marrow involvement.

The following disease presentations were reported in the study group (**Figure 1**):

- Presentation with bone marrow involvement by AML/MDS/CML – 24 patients (55.80%)
- *De novo* presentation in patients without bone marrow involvement **at diagnosis** – 16 patients (37.20%)
- Isolated MS relapse – 1 patient (2.32%)
- Mixed (medullary and extramedullary) relapse of MS – 2 patients (4.65%)

Among patients with CNS involvement, there was one patient (2%) with CNS-2 status and twelve patients (28%) with CNS-3 status. The absence of CNS involvement (CNS-1) was found in twenty-six patients (60%), while in four patients, CNS status was not assessed (no data).

#### Location and Symptoms

Sites of MS in the study group and symptom characteristics are presented in **Table 1**. The most common site was the skin (15 patients, 34.88%), followed by the orbital region (11 patients; 25.58%). Twenty-eight patients (65.11%) had other anatomical areas involved. In ten patients, MS occurred in two or more sites.

**Table 1:** Location of all MS sites in the study group and symptom characteristics.

Location	Symptom	n	n [%]
**Skin (15/24.59%)**	Nodules	**7**	**16.27%**
	Bluish lesions	**1**	**2.32%**
**Orbital region (11/18.03%)**	Exophthalmos	**5**	**11.62%**
	Orbital soft tissue swelling	**2**	**4.65%**
	Ptosis	**1**	**2.32%**
	Facial nerve paralysis	**2**	**4.65%**
	No symptoms (orbital involvement found with imaging tests)	**3**	**6.97%**
**Other (35/57.38%)** Paravertebral area (5/8.19%) Pelvis (3/4.91%) Skull (3/4.91%) CNS (3/4.91%) Lungs (2/3.27%) Pleura (2/3.27%) Abdominal cavity (2/3.27%) Liver (2/3.27%) Middle ear (2/3.27%) Femur (2/3.27%) Cheek (1/1.63%) Elbow joint (1/1.63%) Mammary glands (1/1.63%) Gums (1/1.63%) Nasopharynx (1/1.63%) Intracanal (1/1.63%) Maxillary sinus (1/1.63%) Mediastinum (1/1.63%) Lymph nodes (1/1.63%)	Visible swelling of the affected area/tumor	**8**	**18.6%**
	Fatigue	**8**	**18.6%**
	Pain	**7**	**16.27%**
	Fever	**5**	**11.62%**
	Weight loss	**4**	**9.3%**
	Swollen lymph nodes	**4**	**9.3%**
	7th nerve paralysis	**4**	**9.3%**
	Hearing loss	**3**	**6.97%**
	Symptoms of respiratory tract infection	**3**	**6.97%**
	Paresis	**3**	**6.97%**
	Paleness	**2**	**4.65%**
	Dyspnea	**2**	**4.65%**
	Joint pain	**1**	**2.32%**
	Apathy	**1**	**2.32%**
	Horner’s syndrome	**1**	**2.32%**
	Petechiae	**1**	**2.32%**
	Sweating	**1**	**2.32%**
	Hepatosplenomegaly	**1**	**2.32%**
	Abdominal distension	**1**	**2.32%**
	Constipation	**1**	**2.32%**
	Anuria	**1**	**2.32%**
	Excessive growth of the head circumference	**1**	**2.32%**
	Otitis	**1**	**2.32%**

Number of locations.

A significant relationship was found between the age groups and skin manifestation of MS (skin manifestation was significantly more frequent in patients <10 years of age; **p=0.0268**). In the group of childern with skin manifestation, 7 of 15 (46%) patients were at the age below 12 months.

Symptoms of MS depended on lesion location and the extent of the neoplasm.

#### Genetic Test Results in the Study Group

The most frequent mutation, detected in ten patients (23.25%), was the t(8;21)(q22;q22) translocation. Four patients (9.30%) had KMT2A gene rearrangement. Other aberrations were found in six patients. All the genetic test results of the patients are presented in **Table 2**.

**Table 2:** The results of all genetic tests of bone marrow* in patients from the study group (n=43).

No.	Genetic test result
1	The t(8;21) translocation with AML1/ETO fusion gene on chromosome 8 and loss of the sex chromosome Y, characteristic of AML M2, were found in all metaphases. In addition, complex chromosome aberrations involving chromosomes 11 and 12 were found. BCR/ABL1 - negative. ITD-FLT3 - negative, NPM1 - negative, PML-RARA negative, inv 16 negative.
2	No PML/RARA fusion gene was found, indicating t(15;17) translocation. BCR/ABL fusion gene indicating t(9;22) translocation was found in 13/200 interphase nuclei. No tandem duplication and D85 mutation of the *FLT3* gene were found.
3	In 200 interphase nuclei, no BCR/ABL1 fusion gene was found, indicating t(9;22) translocation. Amplification of the MYCN oncogene over 10 copies was present in 26% of nuclei. 2010-03-02 Karyotype: 46,XY (16) In 200 interphase nuclei, no AML1/ETO fusion gene was found, indicating t(8;21) translocation. In 200 interphase nuclei, no inversion of chromosome 16 was found. No tandem duplication and D85 mutation of the *FLT3* gene were found. In 200 interphase nuclei, no PML/RARA fusion gene was found, indicating t(15;17) translocation. NCN=6400 copies WT1/10 up to 4 copies of ABL.
4	In 90% (180/200) of interphase nuclei, a double AML1/ETO fusion gene was found, indicating t(8;21) translocation. No PML/RARA fusion gene was found, indicating t(15;17) translocation, TEL/AML1, and thus t(12;21), deletion of 11q23, and other rearrangements involving KMT2A, tandem duplication and D85 mutation of the *FLT3* gene, chromosome 5q deletion, BCR/ABL1 fusion gene, and thus the t(9;22) translocation, DEK/NUP214 fusion gene, and thus the t(6;9) translocation. Karyotype: 45 X,Y, t(8;21)(q22;q22).
5*	Normal bone marrow. Tumor tissue tests: *NPM1* positive, 19% of the tumor cells presented 3 signals of the ABL1 I BCR gene.
6	In 10% of interphase nuclei, trisomy of chromosome 8 was found. Cytogenetic analysis (GTG) – normal karyotype in all metaphases. In 40% of interphase nuclei, trisomy of chromosome 8 was found. NMYC amplification – not found.
7	Karyotype: 46 XX. BCR/aBL1 negative, TELAML t(12;21) negative, KMT2A (11q23) negative, *FLT3* negative, inversion 16 - negative, *WT1* – positive.
8	The result of DNA analysis for the most common mutations in the *WT1* and *NPM1* genes – not found. The result of the analysis of the 28 most common chromosomal rearrangements of prognostic significance in leukemia – detection of fusion gene KMT2A-AFF1 t(4;11)(q21;q23).
9	Trisomy 21, the most common chromosome rearrangements in AML – negative.
10	No data
11	Karyotype: 90-92,XXYY,der(4) (15)M-BCR/ABL - negative; KMT2A/AF4 - negative; TEL/AML1 - negative; AML/ETO - negative; m-BCR/ABL - negative; E2A/PBX1 - negative; CBFb-MYH11 - negative; FLT3-ITD. – negative.
12	Karyotype: 45,X,-Y, t (8;21) (14)/46,XY (3); RT-PCR assay: M bcr/abl (-), mbcr/abl (-)
13	t(8;21)(q22;q22)
14	No data
15	t8;21 (q22;q22)
16	No data
17	t(8;21) (FISH + PCR) in bone marrow. *NPM1* and *FLT3* mutations were not found.
18	The bone marrow cytogenetic mutations were identified ins/delins. NPM1 did not detect the translocation of AML 1-ETO and duplication FLT3-ITD. No chromosome aberrations were found.
19	46,XX,der(9)t(9;11;17)(q12;q23;q25),der(11)t(9;11)(p22?;q23),der(17)t(9;17)(q12;q23), del(17)(q23q25). FLT3 (-). KMT2A gene rearrangement on another chromosome was detected in 70% of the analyzed cells.
20	No data
21	No data
22	FLT3(-), WT1(-) NPM1 (-), Hema-Vision 28 N (28 most common rearrangements were not found).
23	46,XX,t(9;11)(9pter~9p22::11q23~11q14.2::9p22~9q32::11q23~11qter;11pter~ 11q14.2::11q2311q23::9q32~9qter) (4)/46,XX (17)
24	t(9;11)(p22;q23)
25	No mitoses, FISH 10% of cells with the KMT2A rearrangement
26	No data
27	No data
28	No data
29	No data
30	No data
31	No data
32	t(8;21)(q22;q22)
33	t(8;21)(q22;q22)
34	Any abnormalities
35	Complex karyotype with clonal evolution
36	t(8;21)(q22;q22)
37	No data
38	30% KOM + t(8;21)(q22;q22)
39	t(8,21)(q22;q22)
40	All negative in AML
41	Abnormal male karyotype with clonal evolution; test performed using the KMT2A-specific probe showed the presence of *KMT2A* gene rearrangement in 93% of the interphase nuclei; metaphases in the sample confirmed the t(9;11)(p21;q23) translocation.
42	No data
43	No data

(*genetic results from tumor tissue in patient 5).

#### Treatment

All the patients (43, 100%) received systemic chemotherapy. In 24 patients (56%), radiotherapy was administered as a part of the treatment; 15 patients (35%) received CNS radiotherapy according to the AML therapeutic regimen, 7 patients (16%) received radiotherapy to the tumor area (with doses of 18-30 Gy), and 2 patients (5%) were irradiated to CNS as well as MS area (with dose of 18 Gy). Six patients (14%) underwent surgical treatment. Nine patients (21%) were treated with alloHSCT. Treatment methods are shown in **Table 3**.

**Table 3:** Methods of MS treatment in the study group.

Treatment method	N	%
**Chemotherapy**	43	100%
**Radiotherapy**	24	56%
*CNS*	15	35%
*tumor area*	7	16%
*CNS and tumor area*	2	5%
**Surgery**	6	14%
*Radical*	5	12%
*Subtotal*	1	2%
**Allogeneic hematopoietic stem cell transplantation**	9	21%

#### Chemotherapy Regimens

First-line chemotherapy regimen was usually based on current treatment guidelines for AML in children; patients were treated according to the AML-BFM 2004 Interim protocol (21 patients, 49%), AML-BFM 2012 protocol (10 patients, 23%), and AML-PPLLSG 98 protocol (10 patients, 23%) (17, 18). Two patients received treatment regimens other than those listed above. Of them, one patient (diagnosed with CML, isolated extramedullary relapse) received imatinib followed by idarubicin in combination with fludarabin (Ida-Fla) and, after complete tumor resection, low-intensity consolidation according to the AML-BFM 2001 protocol while awaiting alloHSCT (17, 19). The second patient, a boy with a relapse of acute bilineal leukemia: T-cell acute lymphoblastic leukemia (T-ALL)/AML and a relapse of AML with MS in the mediastinum, received the treatment according to the Interfant 06 regimen, followed by palliative radiotherapy (20).

In four patients (9.30%) diagnosed with a disease other than MS, a different treatment regimen was used at baseline. Following the accurate diagnosis, the treatment was modified and the following regimens were used:

- for the treatment of lymphoblastic lymphoma (Euro LB 2002) (16) – 1 patient
- for Langerhans cell histiocytosis (HLH 2004) (21) – 1 patient
- for bone sarcoma (VIDE chemotherapy) (22) – 1 patient
- for non-Hodgkin lymphoma (2 x COP prephase according to the Inter-B-NHL Ritux 2010 protocol) (23) – 1 patient

In the second-line treatment, the protocol for AML relapses (Ida-Fla, Fla – 9 patients) was applied most frequently (19), with one patient receiving a regimen with clofarabine (24). Three patients underwent alloHSCT, one of whom had had donor lymphocyte infusion (DLI).

### 2. Treatment Outcomes

In the study group, CR in time was achieved in twenty-nine patients (67%), LR in six patients (14%), and PR in two patients (5%). Five (12%) patients progressed and seven patients (16%) relapsed. Fourteen patients (32%) died due to progression (eleven patients), infectious complications (two patients), and treatment-related toxicity (one patient).

The results of treatment outcomes were as follows (mean ± SD): OS=121.00 ± 13.23 months, pOS=0.67 ± 0.08; RFS=148.09 ± 11.77, pRFS=0.79 ± 0.07; EFS=112.82 ± 13.52, pEFS=0.65 ± 0.08. **Figure 1** shows pOS, pRFS, and pEFS curves.

### 3. Examining the Influence of Selected Factors on Treatment Outcomes

#### Age Group

No differences were found between the age groups (<10 years and >10 years) in OS, RFS, or EFS. Longer OS, RFS, and EFS were observed in younger patients (<10 years), but the differences were not significant (p=0.3584, p=0.8613, and p=0.8613, respectively).

#### Clinical Presentation of MS

**Figure 2:**
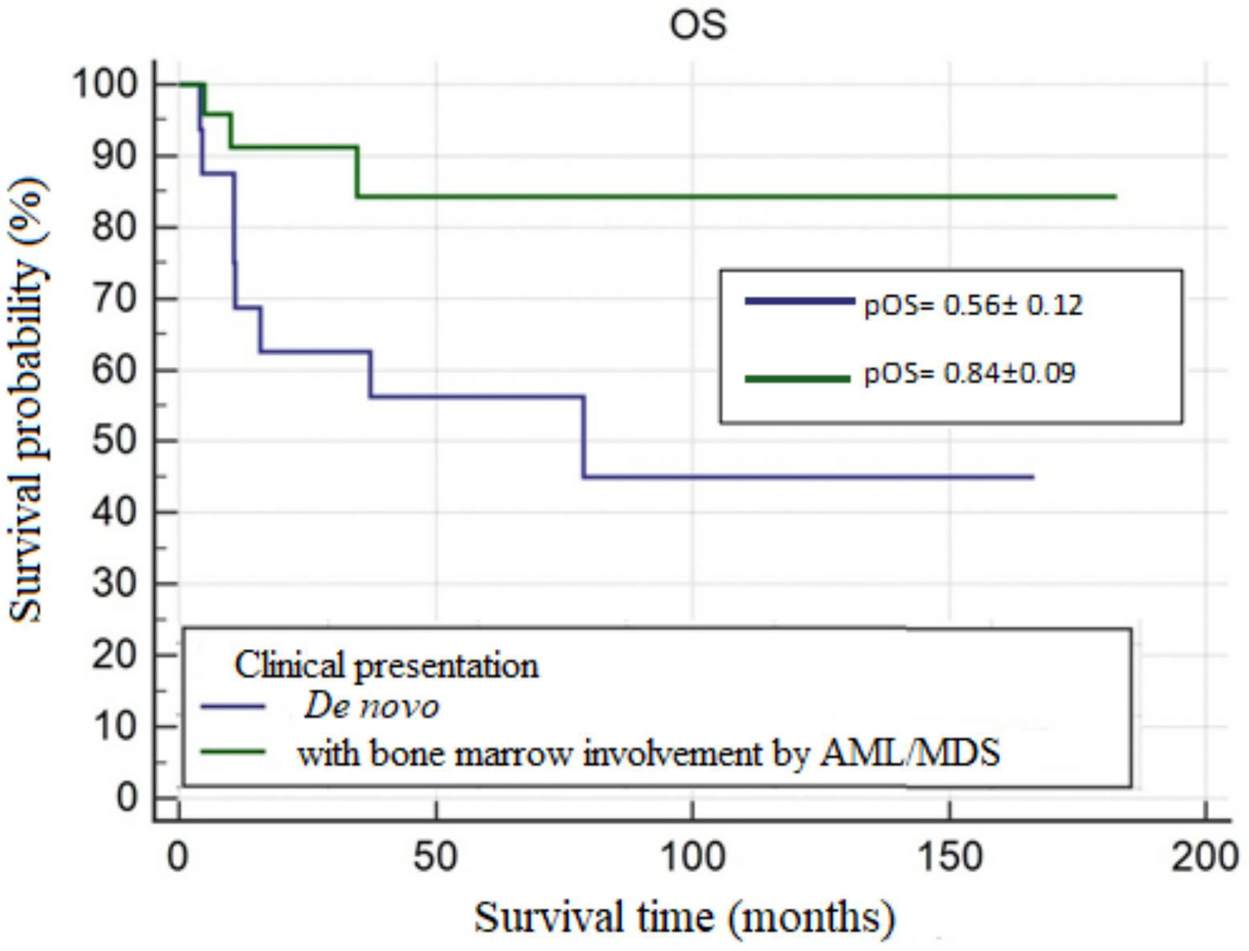
The overall survival probability (pOS) in *de novo* MS and MS with bone marrow involvement by AML/MDS, p=0.0251.

**Figure 3:**
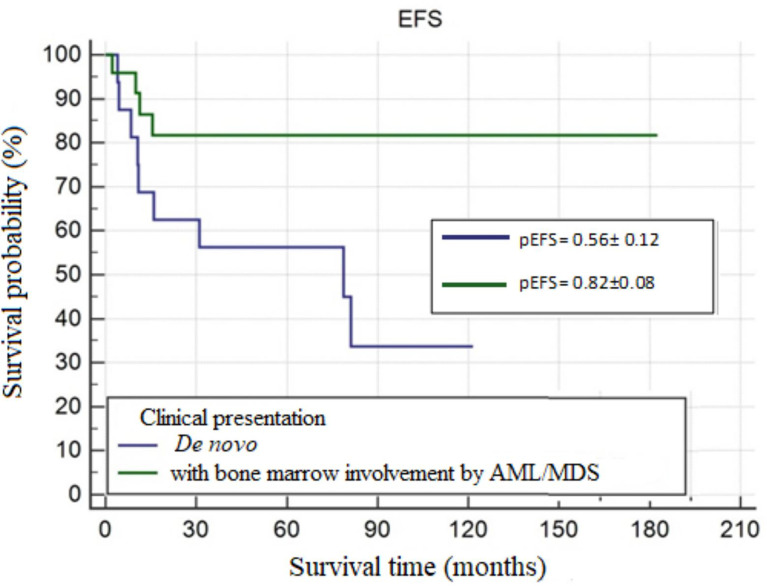
The 5-year event-free survival probability (pEFS) by clinical presentation of MS — *de novo* and MS with bone marrow involvement by AML/MDS, p=.0247.

OS and EFS were significantly longer in patients with MS and concurrent bone marrow involvement by AML/MDS compared to patients with *de novo* MS (**OS**=156.94 ± 13.89 vs. 89.75 ± 19.09; **p=0.0251, EFS**=150.97 ± 14.34 vs. 64.36 ± 12.68; **p=0.0247**). Curves for OS and EFS probability are shown in **Figures 2** and **3**.

#### Location

There were no significant differences in OS, RFS, or EFS between patients with MS in the skin and those with MS in other sites. Patients with the skin presentation of MS had lower pRFS than those with MS involving other sites, although this difference was not significant (0.63 ± 0.16 vs. 0.87 ± 0.07, p=0.0981).

Compared to patients with MS in other sites, those with orbital MS were found to have shorter OS (96.91 ± 17.78 vs. 120.07 ± 15.08) and lower pRFS (0.74 ± 0.16 vs. 0.81 ± 0.08) and pEFS (0.64 ± 0.09 vs. 0.67 ± 0.16). However, the differences were not significant.

#### T(8;21)(q22;q22) Translocation

**Figure 4:**
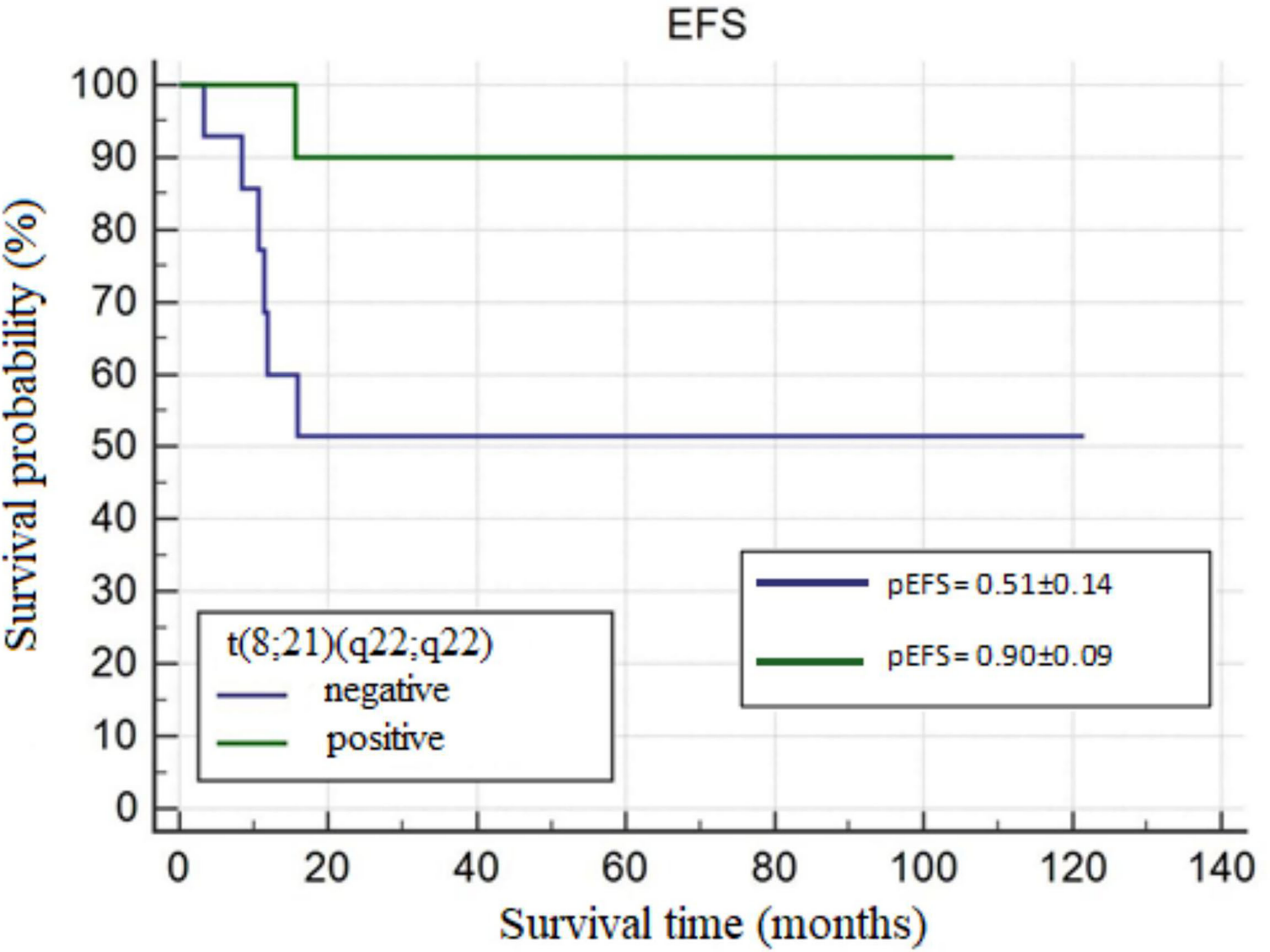
The 5-year event-free survival probability (pEFS) in patients with and without t(8;21)(q22;q22), p=0.0490.

Patients with the t(8;21)(q22;q22) translocation had significantly longer EFS (67.65 ± 15.99 *vs.* 95.25 ± 8.39, **p=0.0490;** **Figure 4** **)** and were more likely to have longer OS (the latter not significant, p=0.0781) compared to those without the t(8;21)(q22;q22) translocation.

#### Treatment Method

The type of therapeutic protocol (AML-PPLLSG, AML-BFM 2004, AML-BFM 2012) did not significantly influence OS, RFS, or EFS.

Longer survival times and higher pOS, pRFS, and pEFS (without significance) were observed in patients who did not undergo alloHSCT.

Neither surgical treatment nor radiotherapy significantly influenced treatment outcomes. Longer OS was found in patients who received radiotherapy (92.97 ± 19.32 vs. 53.88 ± 13.07).

### 4. Relapse Characteristics

Seven patients relapsed, four with a mixed relapse (medullary and extramedullary), two with an isolated extramedullary relapse, and one with an isolated medullary relapse. Out of a total of six patients with extramedullary relapse, five of them had extramedullary sites that were consistent with the primary location of MS. Primary clinical presentation with MS location, time from remission to relapse (months), and relapse location in patients who relapsed are presented in **Table 4**. The shortest time from MS diagnosis to relapse was 2.33 months, while the longest was 182.63 months (median, 31 months).

**Table 4:** Characteristics of MS relapses.

No	Primary MS location	Clinical presentation of MS	Time from remission to relapse (months)	Sites of extramedullary relapse	Bone marrow involvement in relapse [yes/no]
1	Skin	MS with bone marrow involvement (AML)	1 month	Skin	NO (*bone marrow involvement one month after detection of skin involvement)
2	Lung	Isolated extramedullary relapse of CML	15 months	Lung	NO
3	Paravertebral tumor	MS with bone marrow involvement (AML)	12 months	Absence	YES
4	Orbit	MS with bone marrow and CNS involvement (AML, relapse)	11 months	Orbit	YES
5	Abdominal cavity	*De novo*	2 months	Abdominal cavity	NO
6	Skin	MS with bone marrow involvement (AML)	6 months	Orbit and CNS	YES
7	Skin	MS with bone marrow involvement (AML)	10 months	Skin	YES

### 5. Causes of Death

The major cause of death was disease progression (11 patients). Two patients died due to infectious complications, one of whom also experienced treatment-related toxicity. In the group of patients who died, the mean time from diagnosis to death was 18.52 ± 20.24 months (range 3.27-78.67; median, 10.83 months).

## Discussion

### Clinical Characteristics of the Study Group Versus Literature Data

Based on the nationwide database of fourty-three patients diagnosed with MS, the most common clinical presentation was MS accompanying AML, which supports previous literature (3, 5, 9, 10).

As in most reports (25, 26), MS diagnosis usually coincided with AML diagnosis among analyzed patients. There were no asymptomatic patients. However, MS accompanying AML may be underestimated. An analysis by Meyer et al. showed that MS was detected incidentally by imaging studies in 24.5% of patients (27). Stove et al. reported that among 315 children with AML, 39 (12%) were diagnosed with MS at baseline (25). Considering the above findings, it is particularly important that both oncologists and radiologists are aware of this issue.

Isolated MS (*de novo*) is thought to occur less frequently and affect about 1-2% of patients with AML (26). A literature search yielded only two reviews of pediatric patients with isolated MS. In the first one Reinhardt et al. reported 37 children diagnosed with isolated MS in a time frame of 13 years (28). The second study included 10 pediatric patients in Japan (29).

In one patient database, there were as many as 16 patients with *de novo* presentation. In four of them, further diagnostic assessment led to the identification of bone marrow involvement 1 to 2 months after diagnosis.

This study showed that the skin was the most frequent site of MS involvement. Similar findings regarding pediatric patients were reported in analyses available in the literature, including studies by Reinhardt et al. (28), Stove et al. (25), and Dusenbery et al. (11), which demonstrated that the second most common site was the orbital region. Studies including pediatric as well as adult patients revealed different results. The most frequent sites of involvement were lymph nodes (30, 31), followed by the paravertebral region, and then the skin (30).

In the study group, skin manifestation was observed significantly more often in younger patients (under 10 years of age). Based on the studies by Hurley et al., the most common skin lesions were pink or, in some cases, bluish papules (31, 32).

The second most frequent site of MS involvement (n=11, 25.58%) was the orbital region, which is considered a typical site of MS manifestation in children compared to adults, in whom the orbital region is much less frequently involved. Exophthalmos, reported in five patients (11.62%), is a typical and major symptom of orbital MS (33).

Apart from site-related symptoms, the most common symptoms included visible swelling around the lesion/tumor (18.6%), fatigue (18.6%), and pain (16.27%). Patients presented with a variety of symptoms and each patient was a separate and interesting case.

### Summary of Diagnostic Aspects of MS in Children

The gold standard for diagnosis of MS is a tumor biopsy. The histopathological appearance of MS is non-specific and polymorphic; thus, it is vital that the immunohistochemical findings be evaluated in the diagnostic process (34). In this study, twenty-nine patients (67.44%) had tumor biopsy performed. There were patients with isolated MS as well as patients with concurrent bone marrow involvement. Consideration should be given to whether biopsy is necessary in all clinical presentations of MS. According to the literature, biopsy and histopathology should be performed unless the risks of the procedure outweigh its benefits (35).

Four patients (9.30%) were initially diagnosed with another cancer (bone sarcoma, non-Hodgkin lymphoma, and Langerhans cell histiocytosis). Based on the literature, the percentage of diagnostic errors ranges between 25% and 47%, and MS is most frequently misdiagnosed as non-Hodgkin lymphoma (28, 29). This supports the need for a broad panel of antibodies in immunohistochemistry as well as other diagnostic tools.

Flow cytometry provides rapid results of large arrays of antibodies, aiding in diagnosing MS. Therefore, an extensive antibody panel should be performed using fresh tumor tissue provided that it is possible to obtain enough material (36).

A special insight is necessary to diagnose rare *de novo* presentation of MS. Murthy et al. highlighted that a peripheral blood smear should be performed in all patients with exophthalmos and diagnostic evaluation should be expanded to include flow cytometry of bone marrow based on individual indications (33).

Following the case histories contained in the database, to confirm that a patient has isolated MS, the flow cytometry of bone marrow should be performed and, in cases of normal results of aspiration biopsy, trephine biopsy and genetic tests of bone marrow and tumor tissue should be carried out to detect the presence of chromosome rearrangements characteristic of AML. Paraffin blocks for retrospective genetic testing provides valuable insight for MS, phasing out the use fresh samples as a consequence of the time delay in histopathology results and insufficient samples (37). Such tests would be of particular importance in isolated MS and would allow an attempt to classify MS based on the cytogenetic and molecular abnormalities, which would further determine the choice of the most optimal treatment.

In all patients who had PET-CT (4 patients), the scans showed metabolically active sites. It is believed that PET-CT can be an even more sensitive imaging method for MS than computed tomography (25, 27, 38). Because of possible dissemination of MS, PET-CT seems to have additional diagnostic value by imaging all sites of disease (39). In addition, while monitoring treatment outcomes, PET-CT may serve as a more accurate tool for assessing remission, especially if alloHSCT is due to be performed later.

### Treatment Outcomes and Analysis of Selected Prognostic Factors in Children With MS

This study investigated the influence of the following clinical factors on treatment outcomes: patient age (below and above 10 years), MS presentation, MS location, and the presence of t(8;21)(q22;q22) translocation.

#### Age

In this study, longer OS, RFS, and EFS, as well as higher 5-year probability of OS, RFS and EFS, were observed among younger patients (<10 years of age). However, the differences were not significant. In the literature, data on the influence of age on treatment outcomes in patients with MS are extremely limited. The studies by Pileri et al. and Al.-Khaateb et al. showed that age did not influence treatment outcomes (40, 41). Nevertheless, it is known that age is a powerful independent prognostic factor in AML: higher age implies worse prognosis. Lower pOS, accounting for only 50-60%, is observed in adolescents and young adults (AYA) group, i.e., patients aged between 15 and 39 years (42). Bearing in mind that MS usually accompanies AML, perhaps these relationships can be extrapolated to a group of patients with MS.

#### Clinical Presentation

A few studies indicated better treatment outcomes in patients with isolated MS (11, 43–45). This was linked to smaller tumor mass at baseline in those patients compared to patients with MS and bone marrow involvement. By contrast, this study showed that OS **was significantly longer** among patients with MS and **concurrent bone marrow involvement by AML/MDS** compared to patients with *de novo* MS. An analysis by Reinhardt et al. on 34 children with *de novo* MS also reported lower pOS in patients with isolated MS than in those with AML (0.44 ± 0.09 *vs.* 0.55 ± 0.02). Moreover, higher relapse rates were observed among children with isolated MS. Reinhardt et al. highlighted the influence of diagnosis delay on increased risk of relapse (46). However, Pileri et al. did not find any significant differences between the outcomes of patients with isolated MS and those with accompanying AML (40). Unfortunately, reports comparing treatment outcomes of pediatric patients with isolated MS *versus* those with concurrent AML are lacking. Comparisons of treatment outcomes were more often carried out between patients presenting AML without extramedullary involvement and those with AML accompanying MS. Interesting data were published by Pramanik et al., who showed that mean EFS and OS were higher in patients with AML and MS (median EFS was 21.0 months and median OS was 37.1 months) than in those with AML without extramedullary involvement (47).

To conclude, most of the literature considers that isolated MS is associated with poor prognosis. However, it has not been established whether extramedullary disease at AML diagnosis in children is an adverse prognostic factor. It is likely that more powerful factors, e.g., molecular features of cancer cells, determine prognosis.

#### Location

The orbital region and skin, the most common sites of MS involvement in children, have the opposite prognostic significance in the literature. According to the study by Johnston et al., orbital involvement was associated with higher OS compared to patients without extramedullary disease (11). Kobayashi et al. found that extramedullary disease not involving the skin was a favorable prognostic factor (9). In this study, there were no significant differences between OS, RFS, and EFS in patients with MS regarding extramedullary site, including the orbital region. An example of an unfavorable orbital MS course is a patient who presented relapsed orbital MS with concurrent bone marrow involvement.

Manifestation of MS in the skin is considered to be more aggressive and displays a poor prognosis (6, 31, 32). In this study, no significant difference was found between OS of patients with MS of the skin and patients with MS involving other sites (p=0.7017). However, patients with MS of the skin had lower pRFS (p=0.0981). Three patients with skin involvement at baseline relapsed, also in the skin site. This proves that MS of the skin is associated with a high risk of relapse.

#### t(8;21)(q22;q22) Translocation

The t(8;21)(q22;q22) translocation is the most frequently reported cytogenetic abnormality in MS, especially in the pediatric population (5, 36, 48). This was also confirmed by the results of our study. It is known that the t(8;21)(q22;q22) translocation is associated with a favorable prognosis (49). However, data on its prognostic significance in patients with MS are inconclusive. Although Johnston et al. showed that patients with orbital MS had better outcomes, they did not find a relationship between these results and the t(8;21)(q22;q22) translocation (11). Felice et al. found that the t(8;21)(q22;q22) translocation did not worsen treatment outcomes in patients with MS (50). In contrast, Byrd et al. demonstrated that patients with MS and the t(8;21)(q22;q22) translocation experienced worse treatment outcomes, which was associated with more frequent involvement of the meningeal and paraspinal areas (51).

In this study, patients with the t(8;21)(q22;q22) translocation had significantly longer time and probability of EFS, which supports a favorable prognostic value of t(8;21)(q22;q22).

#### Treatment

According to the literature, MS treatment should always be based on systemic polychemotherapy (3, 12, 26). This also applies to patients with isolated MS or with MS after complete surgical resection.

Indeed, apart from patients with MS relapse, all the others from the study group received systemic chemotherapy based on the current treatment regimen for AML. The longest mean OS (146.93 ± 22.25) and the highest pOS (0.80 ± 0.13) were found in patients who were treated with the AML-PPLLSG 98 regimen.

There are neither standards for surgical management and radiotherapy nor indications for alloHSCT in patients with MS, while data published thus far on this subject are ambiguous. In this study, no algorithm for such treatment was found.

Furthermore, there was no significant influence of surgical treatment on OS, RFS, and EFS. However, it is difficult to draw far-reaching conclusions due to a small number of patients; surgery was a part of treatment in six patients (13.95%) from the study group. Patients who did not undergo surgical treatment had longer OS, RFS, and EFS. One patient diagnosed with isolated extramedullary CML relapse (Pancoast tumor) had radical surgery performed to remove the lesion. After 15 months, one patient relapsed in the primary site. Therefore, it is most likely that the key role of surgical treatment is to reduce the symptoms caused by the pressure exerted by the tumor on surrounding structures (12, 35).

Although OS, RFS, and EFS were longer in patients who received radiotherapy, there was also no significant influence of radiotherapy on treatment outcomes. This is in accordance with previous analyses (12, 44). However, the efficacy of radiotherapy with fractionated doses of 20-30 Gy as a part of palliative treatment has been proven in patients with MS (45, 49). Bakst et al. assessed the effects of radiotherapy in 22 patients with MS, indicating that radiotherapy was administered to 90% of the patients for relapse treatment or as a part of first-line treatment in the presence of a residual mass following chemotherapy. Improvement in local symptoms was observed in 95% of patients and treatment was well tolerated (49).

In this study, two patients who received radiotherapy as palliative treatment experienced pain reduction and improvement in symptoms resulting from pressure. The literature describes only single cases of patients in whom radiotherapy was an effective treatment of MS. Yang et al. reported the case of a 19-year-old patient diagnosed with isolated cardiac MS after three years from alloHSCT for AML. Following radiotherapy, the patient improved quickly and achieved remission lasting for six months. Authors put forward a hypothesis that patients post-alloHSCT especially benefit from the radiotherapy due to radiation-induced GvL (52).

Minoia et al. described the case of a 71-year-old woman with MS involving both breasts treated effectively with decitabine and radiotherapy with a dose of 30 Gy (53).

In contrast to some previous reports, no beneficial effect of allotransplantation on outcomes was found in this study (3, 54). Significantly longer RFS was observed in patients who did not undergo hematopoietic stem cell transplantation (p=0.0124). This may arise from the fact that patients with AML and a high risk of relapse and treatment failure are eligible for alloHSCT.

The first-line treatment included alloHSCT in three patients of those who relapsed. Several studies have revealed that the presence of extramedullary disease in patients before alloHSCT is one of the risk factors of extramedullary relapse. To what extent the GvL effect following alloHSCT is effective in extramedullary disease is subject to considerable debate (14, 15). Overall, further broad clinical studies should be done to investigate the role of alloHSCT in the treatment of MS as well as to search for effective methods of prevention and treatment of extramedullary relapse after alloHSCT.

In recent years, chimeric antigen receptor T-cell (CAR-T) therapy has become a revolution in the treatment of myeloproliferative disorders. This therapy uses genetically modified T-cells that acquire the ability to kill a cancer cell. Such type of immunotherapy is already used in the treatment of refractory acute lymphoblastic leukemia and refractory lymphoma. Its use in refractory AML and other myeloproliferative neoplasms is being studied in clinical trials (55). It is unknown how effective it would be in extramedullary disease.

Sorafenib, a kinase inhibitor, offers hope for the treatment of refractory solid tumors and refractory myeloid leukemia in children, especially with the presence of the FLT3-ITD mutation (56). To date, there have been no reports of the use of sorafenib in pediatric patients with MS. Grillo et al. presented a case of an adult patient with extramedullary relapse of AML as MS involving multiple sites after alloHSCT. The patient was successfully treated with sorafenib and subsequent DLI. Genetic tests of tumor tissue revealed the presence of the the FLT3-ITD mutation (56).

Drugs affecting apoptosis may also become a novel option for the treatment of refractory AML. Likewise, inhibitors of BCL-2 (venotoclax), which are already used in the treatment of refractory leukemia in adults, provide hope for improving treatment outcomes in children with refractory myeloid leukemia (57, 58).

## Summary

The evolving nature of revelations of MS warrants specific modern therapeutic regimens. As more and more reports of MS occur due to the enrichment and awareness of MS, it is vital to take all reports into account to examine as a whole the response to current and new treatments in order to prevent relapses, provide optimal treatment, and accurately assess prognosis.

## Data Availability Statement

The data analyzed in this study is subject to the following licenses/restrictions: The dataset was created by the author on the basis of clinical data and patient’s history available in pediatric oncology centers. The author has access to the data which are not public. Requests to access these datasets should be directed to samborska.magda@gmail.com.

## Author Contributions

MS and KD designed the study. JW, ST revised critically the manuscript. MS, MB, JS-S, MC, WBal, SK, KP, MW, TO, TU, GW, JW-T, BU, AC, AK, MK-R, AS-B, IM, MM, AM-M, RT, TS, AC-G, GK, LM-K, NI-J, WBad, MD, PK collected the clinical data. MS drafted the manuscript. KD edited and revised the manuscript. All authors contributed to the article.

## Conflict of Interest

The authors declare that the research was conducted in the absence of any commercial or financial relationships that could be construed as a potential conflict of interest.

## Publisher’s Note

All claims expressed in this article are solely those of the authors and do not necessarily represent those of their affiliated organizations, or those of the publisher, the editors and the reviewers. Any product that may be evaluated in this article, or claim that may be made by its manufacturer, is not guaranteed or endorsed by the publisher.

## References

[1] Burns A. Observations of Surgical Anatomy, Head and Neck (1811) Vol. 1811. Edinburgh: Thomas Royce and Co 1811 364–3.

[2] King A. A Case of Chloroma. Monthly J Med (1853) 1853(17):97.

[3] Avni B Koren-Michowitz M. Myeloid Sarcoma: Current Approach and Therapeutic Options. Ther Adv Hematol (2011) 2(5):309–16. 10.1177/2040620711410774 PMC3573418 23556098

[4] Antic D Elezovic I Milic N Suvajdzic N Vidovic A Perunicic M. Is There a “Gold” Standard Treatment for Patients With Isolated Myeloid Sarcoma? BioMed Pharmacother (2013) 67(1):72–7. 10.1016/j.biopha.2012.10.014 23218987

[5] Ohanian M Faderl S Ravandi F Pemmaraju N Garcia-Manero G Cortes J. Is Acute Myeloid Leukemia a Liquid Tumor? Int. Cancer (2012) 133:534–44. 10.1002/ijc.28012 PMC3904286 23280377

[6] Stefanidakis M Karjalainen K Jaalouk DE Gahmberg CG O'Brien S Pasqualini R. Role of Leukemia Cell Invadosome in Extramedullary Infiltration. Blood (2009) 114(14):3008–17. 10.1182/blood-2008-04-148643 PMC2756207 19636064

[7] Feng S Cen J Huang Y Shen H Yao L Wang Y. Matrix Metalloproteinase-2 and -9 Secreted by Leukemic Cells Increase the Permeability of Blood-Brain Barrier by Disrupting Tight Junction Proteins. PLoS One (2011) 6(8):20599. 10.1371/journal.pone.0020599 PMC3157343 21857898

[8] Faaij CM Willemze AJ Révész T Balzarolo M Tensen CP Hoogeboom M. Chemokine/chemokine Receptor Interactions in Extramedullary Leukaemia of the Skin in Childhood AML: Differential Roles for CCR2, CCR5, CXCR4 and CXCR7. Pediatr Blood Cancer (2010) 55(2):344–8. 10.1002/pbc.22500 20582977

[9] Kobayashi R Tawa A Hanada R Horibe K Tsuchida M Tsukimoto I. Extramedullary Infiltration at Diagnosis and Prognosis in Children With Acute Myelogenous Leukemia. Pediatr Blood Cancer (2007) 48(4):393–8. 10.1002/pbc.20824 16550530

[10] Dusenbery KE Howells WB Arthur DC Alonzo T Lee JW Kobrinsky N. Extramedullary Leukemia in Children With Newly Diagnosed Acute Myeloid Leukemia: A Report From the Children's Cancer Group. J Pediatr Hematol Oncol (2003) 25(10):760–8. 10.1097/00043426-200310000-00004 14528097

[11] Johnston DL Alonzo TA Gerbing RB. Superior Outcome of Pediatric Acute Myeloid Leukemia Patients With Orbital and CNS Myeloid Sarcoma: A Report From the Children’s Oncology Group. Pediatr Blood Cancer (2012) 58(4):519–24. 10.1002/pbc.23201 PMC3165066 21618422

[12] Solh M DeFor TE Weisdorf DJ. Extramedullary Relapse of Acute Myelogenous Leukemia After Hematopoietic Stem Cell Transplantation: Better Prognosis Than Systemic Relapse. Biol Blood Marrow Transplant (2012) 18(1):106–12. 10.1016/j.bbmt.2011.05.023 21703975

[13] Yoo SW Chung EJ Kim SY. Multiple Extramedullary Relapses Without Bone Marrow Involvement After Second Allogeneic Hematopoietic Stem Cell Transplantation for Acute Myeloid Leukemia. Pediatr Transplant (2012) 16(4):125–9. 10.1111/j.1399-3046.2011.01546.x 21923886

[14] Clark WB Strickland SA Barrett AJ. Extramedullary Relapses After Allogeneic Stem Cell Transplantation for Acute Myeloid Leukemia and Myelodysplastic Syndrome. Haematologica (2010) 95(6):860–3. 10.3324/haematol.2010.025890 PMC2878780 20513805

[15] Harris AC Kitko CL Couriel DR. Extramedullary Relapse of Acute Myeloid Leukemia Following Allogeneic Hematopoietic Stem Cell Transplantation: Incidence, Risk Factors and Outcomes. Haematologica (2013) 98(2):179–84. 10.3324/haematol.2012.073189 PMC3561423 23065502

[16] Landmann E Burkhardt B Zimmermann M. Results and Conclusions of the European Intergroup EURO-LB02 Trial in Children and Adolescents With Lymphoblastic Lymphoma. Haematologica (2017) 102(12):2086–96. 10.3324/haematol.2015.139162 PMC5709108 28983060

[17] Balwierz W Pawinska-Wasikowska K Klekawka T. Development of Treatment and Clinical Results in Childhood Acute Myeloid Leukemia in Poland. Memo (2013) 6(1):54–62. 23565126 10.1007/s12254-012-0061-9 PMC3615164

[18] Rasche M Zimmermann M Borschel L. Successes and Challenges in the Treatment of Pediatric Acute Myeloid Leukemia: A Retrospective Analysis of the AML-BFM Trials From 1987 to 2012. Leukemia (2018) 32(10):2167–77. 10.1038/s41375-018-0071-7 PMC6170392 29550834

[19] Yalman N Sarper N Devecioğlu O. Fludarabine, Cytarabine, G-CSF and Idarubicin (FLAG-IDA) for the Treatment of Relapsed or Poor Risk Childhood Acute Leukemia. Turk J Pediatr (2000) 42(3):198–204. 11105617

[20] Brown P. Treatment of Infant Leukemias: Challenge and Promise. Hematol Am Soc Hematol Educ Program (2013) 2013:596–600. 10.1182/asheducation-2013.1.596 PMC4729208 24319237

[21] Bergsten E Horne A Aricó M. Confirmed Efficacy of Etoposide and Dexamethasone in HLH Treatment: Long-Term Results of the Cooperative HLH-2004 Study. Blood (2017) 130(25):2728–38. 10.1182/blood-2017-06-788349 PMC5785801 28935695

[22] Raciborska A Bilska K Drabko K. Validation of a Multi-Modal Treatment Protocol for Ewing Sarcoma–A Report From the Polish Pediatric Oncology Group. Pediatr Blood Cancer (2014) 61(12):2170–4. 10.1002/pbc.25167 25163763

[23] Cairo MS Beishuizen A. Childhood, Adolescent and Young Adult non-Hodgkin Lymphoma: Current Perspectives. Br J Haematol (2019) 185(6):1021–42. 10.1111/bjh.15764 PMC6897376 30729513

[24] Ramos NR Mo C Karp JE. Current Approaches in the Treatment of Relapsed and Refractory Acute Myeloid Leukemia. J Clin Med (2015) 4(4):665–95. 10.3390/jcm4040665 PMC4412468 25932335

[25] Støve HK Sandahl JD Abrahamsson J. Extramedullary Leukemia in Children With Acute Myeloid Leukemia: A Population-Based Cohort Study From the Nordic Society of Pediatric Hematology and Oncology (NOPHO). Pediatr Blood Cancer (2017) 64(12):e26520. doi: 10.1002/pbc.26520 28333413

[26] Lee JY Chung H Cho H. Clinical Characteristics and Treatment Outcomes of Isolated Myeloid Sarcoma Without Bone Marrow Involvement: A Single-Institution Experience. Blood Res (2017) 52(3):184–92. 10.5045/br.2017.52.3.184 PMC5641510 29043233

[27] Meyer HJ Beimler M Borte G. Radiological and Clinical Patterns of Myeloid Sarcoma. Radiol Oncol (2019) 53(2):213–8. 10.2478/raon-2019-0014 PMC6572499 30893056

[28] Reinhardt D Creutzig U. Isolated Myelosarcoma in Children – Update and Review. Leuk Lymphoma (2002) 43(3):565–74. 10.1080/10428190290012056 12002760

[29] Taga T Imamura T Nakashima K. Clinical Characteristics of Pediatric Patients With Myeloid Sarcoma Without Bone Marrow Involvement in Japan. Int J Hematol (2018) 108(4):438–42. doi: 10.1007/s12185-018-2492-5 29971602

[30] Kawamoto K Miyoshi H Yoshida N. Clinicopathological, Cytogenetic, and Prognostic Analysis of 131 Myeloid Sarcoma Patients. Am J Surg Pathol (2016) 40(11):1473–83. 10.1097/PAS.0000000000000727 27631510

[31] Cho-Vega JH Medeiros LJ Prieto VG. Leukemia Cutis. Am J Clin Pathol (2008) 129(1):130–42. 10.1309/WYACYWF6NGM3WBRT 18089498

[32] Hurley MY Ghahramani GK Frisch S. Cutaneous Myeloid Sarcoma: Natural History and Biology of an Uncommon Manifestation of Acute Myeloid Leukemia. Acta Derm Venereol (2013) 93(3):319–24. 10.2340/00015555-1458 23165700

[33] Murthy R Vemuganti GK Honavar SG. Extramedullary Leukemia in Children Presenting With Proptosis. J Hematol Oncol (2009) 24:2–4. 10.1186/1756-8722-2-4 PMC2651909 19166619

[34] Almond LM Charalampakis M Ford SJ. Myeloid Sarcoma: Presentation, Diagnosis and Treatment. Clin Lymphoma Myeloma Leuk (2017) 17(5):263–7. 10.1016/j.clml.2017.02.027 28342811

[35] Bakst RL Tallman MS Douer D. How I Treat Acute Myeloid Leukemia. Blood (2011) 118:3785–93. 10.1182/blood-2011-04-347229 21795742

[36] Wilson CS Medeiros LJ. Extramedullary Manifestations of Myeloid Neoplasms. Am J Clin Pathol (2015) 144:219–39. 10.1309/AJCPO58YWIBUBESX 26185307

[37] Mirza Kamran M Sukhanowa M Stolzel F. Genomic Aberrations in Myeloid Sarcoma Without Blood or Bone Marrow Involvement: Characterization of Formalin-Fixed Paraffin-Embedded Samples by Chromosomal Microarrays. Leuk Res (2014) 38:1091–6. 10.1016/j.leukres.2014.05.004 PMC4157130 25088808

[38] Stolzel F Röllig C Radke J. F-FDG-PET/CT for Detection of Extramedullary Acute Myeloid Leukemia. Haematologica (2011) 96(10):1552–6. 10.3324/haematol.2011.045047 PMC3186319 21685468

[39] Chandra P Dhake S Purandare N. Role of FDG PET/CT in Diagnostic Evaluation of Granulocytic Sarcomas: A Series of 12 Patients. Indian J Nucl Med (2017) 32(3):198–202. 28680203 10.4103/ijnm.IJNM_10_17 PMC5482015

[40] Pileri SA Ascani S Cox MC. Myeloid Sarcoma: Clinico-Pathologic, Phenotypic and Cytogenetic Analysis of 92 Adult Patients. Leukemia (2007) 21:340–50. 10.1038/sj.leu.2404491 17170724

[41] Al-Khateeb H Badheeb A Haddad H. Myeloid Sarcoma: Clinicopathologic, Cytogenetic, and Outcome Analysis of 21 Adult Patients. Leuk Res Treat (2011) 2011:523168. 10.4061/2011/523168 PMC3505919 23213544

[42] Creutzig U Buchner T Sauerland MC. Significance of Age in Acute Myeloid Leukemia Patients Younger Than 30 Years: A Common Analysis of the Pediatric Trials AML-BFM 93/98 and the Adult Trials AMLCG 92/99 and AMLSG HD93/98A. Cancer (2008) 112:562–71. 10.1002/cncr.23220 18076087

[43] Movassaghian M Brunner AM Blonquist TM. Presentation and Outcomes Among Patients With Isolated Myeloid Sarcoma: A Surveillance, Epidemiology, and End Results Database Analysis. Leuk Lymphoma (2015) 56(6):1698–703. 10.3109/10428194.2014.963080 25213180

[44] Lan TY Lin DT Tien HF. Prognostic Factors of Treatment Outcomes in Patients With Granulocytic Sarcoma. Acta Haematol (2009) 122(4):238–46. 10.1159/000253592 19887783

[45] Chen WY Wang C Chang CHH. Clinicopathologic Features and Responses to Radiotherapy of Myeloid Sarcoma. Radiat Oncol (2013) 8:245. 24148102 10.1186/1748-717X-8-245 PMC4016483

[46] Reinhardt D Pekrun A Lakomek M. Primary Myelosarcomas Are Associated With a High Rate of Relapse: Report on 34 Children From the Acute Myeloid Leukaemia-Berlin-Frankfurt-Münster Studies. Br J Haematol (2000) 110(4):863–6. 10.1046/j.1365-2141.2000.02290.x 11054069

[47] Pramanik R Tyagi A Chopra A. Myeloid Sarcoma Predicts Superior Outcome in Pediatric AML; Can Cytogenetics Solve the Puzzle? Clin Lymphoma Myeloma (2018) 18(6):249–54. 10.1016/j.clml.2018.03.013 29680411

[48] Di Veroli A Micarelli A Cefalo M. Recurrence of a T(8;21)-Positive Acute Myeloid Leukemia in the Form of a Granulocytic Sarcoma Involving Cranial Bones: A Diagnostic and Therapeutic Challenge. Case Rep Hematol (2013) 2013:245395. 24109526 10.1155/2013/245395 PMC3787645

[49] Bakst R Wolden S Yahalom J. Radiation Therapy for Chloroma (Granulocytic Sarcoma). Int J Radiat Oncol Biol Phys (2012) 82(5):1816–22. 10.1016/j.ijrobp.2011.02.057 PMC5045241 21962486

[50] Felice MS Zubizarreta PA Alfaro EM. Good Outcome of Children With Acute Myeloid Leukemia and T(8;21)(Q22;Q22), Even When Associated With Granulocytic Sarcoma: A Report From a Single Institution in Argentina. Cancer (2000) 88(8):1939–44. 10760772

[51] Byrd JC Weiss RB Arthur DC. Extramedullary Leukemia Adversely Affects Hematologic Complete Remission Rate and Overall Survival in Patients With T(8;21)(Q22;Q22): Results From Cancer and Leukemia Group B 8461. J Clin Oncol (1997) 15(2):466–75. 10.1200/JCO.1997.15.2.466 9053467

[52] Yang WC Yao M Chen YH. Complete Response of Myeloid Sarcoma With Cardiac Involvement to Radiotherapy. J Thorac Dis (2016) 8(6):1323–8. 10.21037/jtd.2016.04.47 PMC4885973 27293853

[53] Minoia C De Fazio V Scognamillo G. Long-Lasting Remission in *De Novo* Breast Myeloid Sarcoma Treated With Decitabine and Radiotherapy. Diagnostics (Basel) (2019) 9(3):84. 10.3390/diagnostics9030084 PMC6787642 31357576

[54] Chevallier P Labopin M Cornelissen J. Allogeneic Hematopoietic Stem Cell Transplantation for Isolated and Leukemic Myeloid Sarcoma in Adults: A Report From the Acute Leukemia Working Party of the European Group for Blood and Marrow Transplantation. Haematologica (2011) 96(9):1391–4. 10.3324/haematol.2011.041418 PMC3166114 21685467

[55] Mardiana S Gill S. CAR T Cells for Acute Myeloid Leukemia: State of the Art and Future Directions. Front Oncol (2020) 10:697. 32435621 10.3389/fonc.2020.00697 PMC7218049

[56] Grillo G Zucchetti E Forno B. Targeted Therapy in FLT3-ITD Positive Mieloid Sarcoma: Proof of Principle. Blood (2017) 130:5061.

[57] Karol SE Cooper T Bittencourt H. Safety, Efficacy, and PK of the BCL2 Inhibitor Venetoclax in Combination With Chemotherapy in Pediatric and Young Adult Patients With Relapsed/Refractory Acute Myeloid Leukemia and Acute Lymphoblastic Leukemia: Phase 1 Study. (2019) 134(Supplement_1):2649.

[58] Campidelli C Agostinelli C Stitson R. Myeloid Sarcoma: Extramedullary Manifestation of Myeloid Disorders. Am J Clin Pathol (2009) 132(3):426–37. 10.1309/AJCP1ZA7HYZKAZHS 19687319

